# Pickering emulsion gel stabilized by milk fat globule membrane/pectin enhanced probiotic stability

**DOI:** 10.1016/j.fochx.2025.102409

**Published:** 2025-03-24

**Authors:** Yu Ji, Yifan Wu, Yan Wang, Shuangshuang He, Yishan Jiang, Xin Li, Liupeng Wang, Qian Xu, Lili Zhang, Hao Wang

**Affiliations:** a Key Laboratory of Dairy Science, College of Food Science*,* Northeast Agricultural University*,* Harbin 150030*,* China; b College of Food Science and Engineering, Tarim University, Alaer 843300, China; c College of Life Science, Northeast Agricultural University, Harbin 150030, China

**Keywords:** Milk fat globule membrane, Pectin, Pickering emulsion, Probiotics, Encapsulation

## Abstract

The milk fat globule membrane (MFGM) has been shown to improve probiotic survival in the gastrointestinal tract. However, emulsions stabilized through electrostatic interactions between MFGM and polysaccharides for probiotic encapsulation remain unexplored. This study established optimal conditions for creating MFGM-Pectin (CP) emulsions. At pH 4.0, a MFGM/CP ratio of 1 produced the smallest particle size and the highest zeta potential, ideal for emulsion stabilization. We evaluated the effects of varying complex concentrations and oil phase ratios on emulsion properties. The results indicate that CP significantly affects the apparent viscosity, oxidative stability, centrifugal stability, storage stability, and antioxidant activity of MFGM-CP emulsions. Furthermore, the encapsulation of LGG within the emulsion improved its survival and storage stability in simulated gastrointestinal fluids. These findings suggest that MFGM is a promising material for probiotic encapsulation and provide a foundation for developing MFGM-based products containing probiotics or other active ingredients.

## Highlights

• Pickering emulsions was prepared by MFGM and CP particles.
• CP Supplementation enhanced the storage stability of MFGM emulsions.
• MFGM-CP enhanced probiotic survival in gastrointestinal fluids.
• MFGM-CP improved probiotic storage stability.

## 1 Introduction

In recent years, Pickering emulsions have emerged as a focal point in food research due to their unique structure, superior stability and safety as food-grade emulsions (Anjali & Basavaraj, 2018). Their potential applications include animal fat substitutes (Gao et al., 2023), carriers for bioactive substances (Liang et al., 2023), and smart food films (Xu et al., 2023). Unlike conventional emulsions, Pickering emulsions utilize solid particles as emulsifiers, excluding low molecular weight surfactants. Their fundamental structure comprises an oil phase, an aqueous phase and solid particles, which can be categorized as simple solid particles (polysaccharide particles-protein particles) and complex solid particles (protein-polysaccharide, protein-polyphenol, ternary complex particles, and fat crystals) (Xia et al., 2021). However, limited research has examined whether proteins containing lipids or polar lipid components can form Pickering emulsions.

Milk fat globule membrane (MFGM), a unique three-layered structure surrounding the milk fat globule containing 30–75% polar lipids — primarily glycerophospholipids and sphingolipids (Raza et al., 2021), and 25–75% proteins, including butyrophilin (BTN), mucin 1 (MUC1), xanthine oxidoreductase (XDH/XO), and fatty acid binding proteins (Lopez et al., 2017). MFGM also contains minor components like cholesterol, glycolipids, and enzymes that are vital for maintaining its structure and function (Nie et al., 2024). Its natural emulsifying properties have led to its investigation primarily as an emulsifying agent in infant formulas (Chitchumroonchokchai et al., 2023). Additionally, MFGM can enhance the survival of probiotics in the gastrointestinal tract (Zhang, et al. 2020), warranting its exploration as a stable encapsulation matrix for probiotics. The surface active and emulsifying proteins and the phospholipids in MFGM can promote oil-water mixing and stabilize emulsions (Yu et al., 2022). Special components of MFGM may also provide physiological benefits, including anti-inflammatory and anti-aging properties (Li et al., 2020).

Pectin (CP) is a naturally occurring polysaccharide widely employed as a thickener, emulsifier and stabilizer in food products (Assadpour et al., 2017). Research indicates that CP can interact with proteins via electrostatic interactions to form Pickering emulsions without traditional surfactants (Jiang et al., 2020; Wang et al., 2023). Compared with traditional protein-polysaccharide emulsions, the phospholipid layer of MFGM offers enhanced superior thermal stability, facilitating the targeted delivery of heat-sensitive bioactives (e.g., probiotics, polyphenols). In addition, MFGM has been shown to improve probiotic bile salt tolerance and intestinal colonization. Thus, we investigated whether emulsions prepared with MFGM improve the storage stability and intestinal tolerance of probiotics. However, no research has explored the potential for CP to engage in non-covalent interactions with MFGM-containing lipids to form emulsions. We hypothesized that the synergistic interaction between MFGM and CP can harness their respective advantages to develop a stable emulsification system and a high-quality Pickering emulsion for probiotic encapsulation.

Probiotics are known for their health-promoting effects, including relieving inflammatory bowel disease (Zhou et al., 2022), lowering cholesterol, regulating intestinal flora, and enhancing immunity (Thakkar et al., 2016). However, their bioavailability is often hindered by poor resistance to adverse conditions, leading to reduced viability during production, transport, and gastrointestinal digestion (Huang et al., 2024). Therefore, researchers are increasingly focused on developing effective probiotic delivery systems, employing methods such as lyophilization, electrostatic spinning, coating, and emulsification (Xie et al., 2023). Encapsulation technology and material greatly influence probiotics viability (Terpou et al., 2019), Emulsion systems offer benefits such as ease of handling, suitability for large-scale production, and flexibility for modification through coating and gelation. Research indicates that dispersing probiotics within the oil phase of emulsions protects them from air and moisture, thereby preserving their activity. Consequently, emulsions are increasingly recognized as effective probiotic delivery systems (Qin et al., 2021).

The study aims to verify the feasibility of MFGM and CP as Pickering emulsion stabilizers to enhance probiotic stability. We investigated the structural characteristics and macroscopic properties of various MFGM-CP concentrations (c, 0.5–4%) and oil phase ratios (ϕ, 0.1–0.3). Additionally, we assessed the storage viability and survival of *Lactobacillus rhamnosus* GG (LGG) in MFGM-CP emulsions after simulated gastrointestinal digestion. Our findings expand the application potential of MFGM and provides theoretical basis for developing MFGM-based products and improving probiotic processing, storage stability and intestinal tolerance.

## 2 Materials and methods

### 2.1 Materials

Milk Fat Globule Membrane-10 (MFGM, 69–76% protein, 16–22% fat) was purchased from Arla Food Ingredients Group (Viby, Denmark). Pectin (CP, citrus source, 65% esterification) was purchased from Yuanye Biotechnology Co. Ltd. (Shanghai, China). Rice oil and *Lactobacillus rhamnosus* GG (LGG) (ATCC 53103) were purchased from Kerry Food Industry Co. Ltd. (Shanghai, China) and China center of industrial culture collection (Beijing, China), respectively. Other reagents were obtained from Solarbio Technology Co. Ltd. (Beijing, China) or McLean Biochemical Technology Co. Ltd. (Shanghai, China).

### 2.2 Preparation of MFGM-CP complexes

One gram of either MFGM or CP were dissolved in sterile deionized water to create 1% (*w*/*v*) solution, stirred at 800 rpm for 2 h, and refrigerated at 4 °C overnight for protein hydration. MFGM-CP complex solutions were prepared by mixing the MFGM and CP solution in various ratios (1,1, 1,2, 1,3, 3,1, 2:1, *w*/w), adjusting pH with 0.1 mol/L HCl and 0.1 mol/L NaOH, and then stirred at 1000 rpm for 30 min at 25 °C. The final solution was stored at 4 °C for later use.

### 2.3 Zeta potentials determination of MFGM-CP complexes

Zeta potentials of MFGM, CP, and MFGM-CP complex solutions (1,1、1,2、1,3、3,1、2:1, w/w) across pH 2.0–7.0 were determined using a Zetasizer Nano ZS90 (Malvern Instruments Ltd., Worcestershire, UK) (Dong et al., 2023). One mL of each sample was placed in the test cell, at 25 °C.

### 2.4 Particle size analysis of MFGM-CP complexes

MFGM-CP complex solutions (1,1, 1,2, 1:3, 3:1, 2:1, w/w) at pH 4.0 were stored at room temperature for 24 h. Deionized water at pH 4.0 served as a dispersant to dilute the MFGM-CP complex solutions (1 mg/mL) to reduce data variability from multiple scattering. The Zetasizer Nano ZS90 was employed to determine the average particle size and polydispersity index (PDI), with a sample refractive index set to 1.33 (Wei et al., 2019).

### 2.5 Preparation of particle-stabilized Pickering emulsions of MFGM-CP complexes

After a comprehensive analysis of the zeta potential, PDI, and particle size data, MFGM-CP complexes (1:1, at pH 4.0) were identified as potential Pickering stabilizers. The preparation of these complexes as Pickering stabilizers was shown as follows. Pickering emulsions stabilized by MFGM-CP complexes were prepared by mixing MFGM-CP (1:1) complex solutions of various concentrations (c, 0.5%, 1%, 2%, 3%, 4%, *w*/*v*) with oil phases at varying ratios (ϕ, 0.1, 0.2, 0.3). MFGM-CP complex particles were dispersed in sterile deionized water (aqueous phase) prior to adding rice oil (oil phase). MFGM-CP Pickering emulsions (MCP) were processed using a high-speed homogenizer (T18 Digital ULTRA-TURRAX, IKA, Germany) at 15,000 rpm for 3 min.

### 2.6 Types of MCP emulsions and observations by light microscopy

The MCP emulsion (50 μL) prepared in 2.5 was dropped into 5 mL pure rice oil and pure water, respectively. Emulsions dispersed rapidly in water but remained aggregated in oil were classified as O/W (Wei & Huang, 2019). Droplets were observed on microscope slides at 400 × magnification at 25 °C (Qin et al. 2021).

### 2.7 Emulsification properties of MCP emulsions

The emulsification activity index (EAI) and emulsion stability index (ESI) were assessed following Yan et al.'s method (Yan et al., 2024). Emulsion (50 μL) was mixed with 4.95 mL of sodium dodecyl sulfate (SDS) (0.1 g/L), and absorbance was measured at 500 nm. EAI and ESI were calculated as follows.

$$\mathrm{EAI}\;\left(m^{2}/g\right)=2\times 2.303\frac{A_{0}\times N}{\rho \times \phi \times 10000};$$

$$\mathrm{ESI}\;\left(\min\right)=\frac{A_{0}\times 30}{A_{0}-A_{30}};$$

where A_0_ is the initial absorbance of the mixture; A_30_ is the absorbance after 30 min; N is the dilution factor (100); ρ is the emulsion concentration (g/mL); ϕ is the oil phase volume fraction.

### 2.8 Determination of viscosity properties of MCP emulsions

Viscosity properties of emulsions were evaluated using a rotational rheometer (HAAKE MARS40, Thermo Electron Corp., Germany). Viscosity was measured over a shear rate range of 0.01 to 200 s^−1^ (Dong et al., 2023).

### 2.9 Storage stability of MCP emulsions

Emulsions were stored at 4 °C for one month. The total height (Ht) and cream height (Hc) of the emulsions were recorded before and after storage (samples taken at 1, 2, 3, 5, 7, 14, 21, and 30 d), and the creaming index (CI) was calculated using the formula (Hc/Ht) × 100% (Dong et al., 2023).

### 2.10 Oxidative stability of MCP emulsions

Emulsions were placed in 50 mL centrifuge tubes and stored at 50 °C for 15 days, protected from light. Lipid oxidation was determined by lipid hydroperoxide (LH) and thiobarbituric acid reactive substances (TBARS) analyses at days 0, 3, 6, 9, 12, and 15 (Pan et al., 2020).

For LH analysis, 0.3 mL of the emulsion was mixed with 1.5 mL isooctane/2-propanol (3:1, v: v) and vortexed for 1 min. The upper organic phase (0.2 mL) was collected by centrifugation at 4000 *g* for 3 min and mixed with 2.8 mL of methanol/1-butanol (2:1, v: v), and then 15 μL each of 3.94 mol/L ammonium thiocyanate and ferrous chloride solution (from 0.144 mol/L FeSO_4_ and 0.132 mol/L BaCl_2_) were added. The mixture was reacted in the dark for 20 min, with absorbance measured at 510 nm. The hydroperoxide concentration was calculated from a hydroperoxyisopropylbenzene standard curve.

For TBARS analysis, 1 mL of sample was combined 5 mL of TBA solution (0.375% thiobarbituric acid, 15% trichloroacetic acid in 0.25 mol/L HCl) and heated at 100 °C for 15 min. The mixture was then cooled and filtered through a 0.22 μm membrane. Absorbance was measured at 532 nm, and TBARS concentration was calculated using a standard curve established with 1,1,3,3-tetraethoxypropane.

### 2.11 Centrifugal stability of emulsions

The effects of temperature and salt concentration on emulsion stability were determined as a previous method (Yan et al., 2024). Briefly, 100 μL of the emulsion was diluted in 5 mL of deionized water and absorbance was measured at 500 nm. The centrifugal stabilization constant (Ke) was calculated as Ke (%) =$\frac{A_{1}}{A_{0}}$, where A_0_ and A_1_ represent absorbance before and after centrifugation at 3000 *g* for 15 min, respectively.

### 2.12 Measurement of DPPH free radical scavenging capacity of emulsions

The DPPH free radical scavenging capacity of emulsions was determined according to the method of Han et al. (2024). Emulsions at 2 mg/mL with varying oil-to-water ratios (ϕ, 0.1–0.3%) were mixed with DPPH (0.2 mmol/L in ethanol) in a 1:1 (*v*/v) ratio, stored in the dark for 30 min, and absorbance (A_t_) was recorded at 517 nm. Scavenging activity (%) was calculated using the following equation:

$$\text{Scavenging activity}\;\left(\%\right)=(1-\frac{A_{t}-A_{b}}{A_{c}})\;\times 100\;\%$$

where A_b_ is the absorbance of DPPH-ethanol replaced by ethanol, and A_c_ is the absorbance of the DPPH-ethanol and deionized water mixture.

### 2.13 ABTS free radical scavenging capacity of emulsions

The scavenging capacity of ABTS free radical in emulsions was determined using Liu et al.'s method (Liu et al., 2020). An ABTS solution (7.0 mmol/L) was mixed with potassium persulfate solution (4.9 mmol/L) in a 1:1 (v/v) ratio and kept in the dark for 16 h at 20 °C. The mixture was diluted until its absorbance at 734 nm reached 0.70 ± 0.02. A 0.2 mg/mL sample was then mixed 1:1 with diluted ABTS solution and incubated for 30 min, measuring absorbance at 734 nm (A_t_), with phosphate buffer solution (PBS) as a blank. Scavenging activity (%) was calculated using the following equation:

$$\text{Scavenging activity}\;\left(\%\right)=(1-\frac{A_{t}-A_{b}}{A_{c}})\;\times 100\;\%$$

where A_b_ represents the absorbance of the PBS solution, and A_c_ denotes the absorbance of the mixture of PBS and ABTS.

### 2.14 Embedding LGG in Pickering emulsion

LGG was cultured according to previous reports (Wang et al., 2024). The 30 mL of bacterial culture was centrifuged at 7000 *g* for 5 min at 4 °C, washed twice with equal volume of 0.85% (*w*/*v*) saline, and resuspended in 3, 6, and 9 mL of sterile rice oil, respectively. Then, 27, 24, and 21 mL of 4% sterile MFGM-CP were mixed with bacterial cell oil solution to create MCP emulsions with ϕ values (ϕ,0.1, 0.2, and 0.3), using shear at 15,000 rpm for 3 min, for embedding LGG in Pickering emulsion, which was stored at 4 °C for subsequent analyses. Unencapsulated LGG, appropriately diluted, served as a control.

### 2.15 Storage stability of LGG

Unencapsulated LGG and Pickering emulsions containing LGG (c = 4%, ϕ of 0.1, 0.2, and 0.3) were stored at 4 °C for 28 d, designated MCP-1, MCP-2, and MCP-3 based on ϕ values. Live LGG release was performed by centrifuging samples at 15,800 g for 10 min (Liang et al., 2023). Samples were collected on days 0, 7, 14, 21, and 28, with surviving LGG quantified after 24 h incubation at 37 °C using the drop plate method (Wang et al., 2024). Free LGG in sterile water served as a control.

### 2.16 Digestive stability of LGG in simulated gastrointestinal fluids

Simulated gastric fluid (SGF) was prepared according to Qin et al. (2021) with modifications, using 85 mmol/L NaCl and 3 g/L pepsin, at pH 2.0, 1.8, and 1.5. Similarly, simulated intestinal fluid (SIF) was prepared with 85 mmol/L NaCl, 10 mmol/L CaCl_2_, 1 g/L bile salts, and 1 g/L pancreatic enzymes, and pH was adjusted to 7.0 with 1.0 mol/L NaOH. LGG-embedded Pickering emulsion was mixed with SGF and SIF (1:1) and incubated at 150 rpm on a shaker at 37 °C for 3 h. Samples were taken hourly, serially diluted, and incubated on MRS agar plates for 24 h to measure viable cell counts. Free LGG in sterile water was also used as a control.

### 2.17 Data analysis

All experiments were performed independently in triplicate and results are expressed as mean ± standard deviation (±SD). Graphs were drawn using Origin 2021Pro. Data were analyzed by ANOVA with SPSS software, and significance was analyzed using the Waller-Duncan method (*P* < 0.05).

## 3 Results and discussion

### 3.1 Zeta potential and particle size of MFGM-CP complexes

**Fig. 1:**
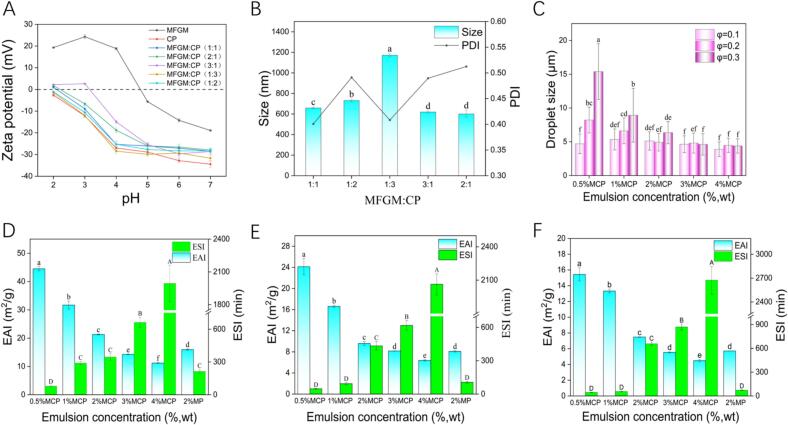
Properties of MFGM-CP solutions and complexes. (A) Zeta potentials of MFGM, CP, and MFGM-CP solution at varying ratios; (B) average particle size and polydispersity index (PDI) of MFGM-CP complexes across different ratios; (C) average droplet sizes of emulsions stabilized by various concentrations of MFGM-CP complexes (c, 0. 5–4%) and oil phase ratios (ϕ, 0.1–0.3); (D—F) Emulsion activity index (EAI) and emulsion stability index (ESI) of MCP emulsions at ϕ = 0.1, ϕ = 0.2 and ϕ = 0.3. Upper and lower case letters represent statistical differences in emulsion ESI and EAI, respectively. Different letters indicate significant differences (*P* < 0.05).

The absolute value of the zeta potential reflects solution stability; a larger absolute value indicates greater stability (Dong et al., 2023). To demonstrated the electrostatic nature of complexation between MFGM and CP, we analyzed their zeta potentials. Fig. 1A illustrates how the zeta potentials of MFGM, CP, and MFGM-CP complexes vary with pH. In the pH range of 2.0–7.0, MFGM's zeta potential shifted from positive to negative, reaching an isoelectric point at pH 4.7, while CP remained negatively charged, leading to a gradually decrease in the zeta potential of MFGM-CP complex. The greatest difference between MFGM's positive zeta potential and CP's negative zeta potential occurred at pH 4.0, deemed optimal for their complexation (Estrada-Fernandez et al., 2018). However, a high concentration of CP may induce a charge shielding effect (Liang et al., 2023), similar to occurrences in chitosan interactions with perilla protein (Zhao, et al. 2023). Thus, MFGM-CP complexes at varying ratios (1:1, 1:2, 1:3, 3:1, 2:1, *w*/w) at pH 4.0 were prepared for further analysis.

Particle size is influenced by various interactions (e.g., electrostatic interactions, hydrophobic interactions), with smaller particles enhancing droplet interface coverage and emulsion stability (Wu & Ma, 2016). Fig. 1B presents the particle size and PDI of MFGM-CP complexes at pH 4.0. Increasing CP concentration resulted in larger average particle sizes, yet zeta potential remained constant, potentially due to weaker electrostatic repulsion compared to hydrophobic interaction and hydrogen bonding. The smallest particle size was recorded for 1:1, 2:1 and 3:1 ratio, with the 1:1 ratio yielding a PDI of 0.4008. A lower PDI indicates a more homogeneous dispersion with minimal size variation (Su et al., 2018). Thus, optimal MFGM-CP complex preparation occurs at a 1:1 mass ratio and pH 4.0. Fig. 8 illustrates the formation of MFGM-CP complexes, where the positively charged MFGM and the negatively charged CP were bound together by electrostatic mutual attraction at pH 4.0.

### 3.2 Microstructural analysis of MCP emulsions

**Fig. 2:**
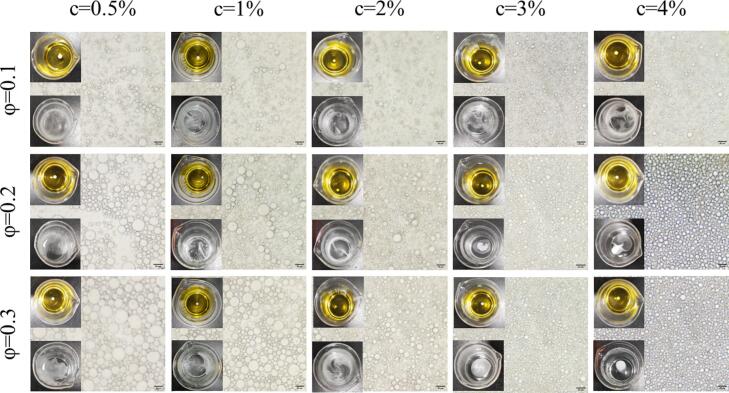
Visual observation and representative optical microscope images of fresh emulsions prepared with varying concentration of MFGM-CP complex (c, 0.5–4%, *w*/*v*) and oil phase ratios (ϕ, 0.1–0.3).

Fig. 2 illustrates the microstructures of emulsions at various MFGM-CP complex concentrations (c, 0.5–4%, *w*/*v*) and oil phase ratios (ϕ, 0.1–0.3). Emulsion droplets appeared spherical under microscopy, but emulsions with lower concentrations (0.5–2%) were unstable and susceptible to delamination due to droplet aggregation. The average size of the emulsion droplets was measured using Image J software and the results showed that the increasing MFGM-CP complex concentration from 0.5% to 4.0% significantly reduced droplet size (Fig. 1C), with average sizes decreasing from 15.39 ± 4.12 μm to 4.39 ± 1.06 μm at ϕ = 0.3. Limited particle numbers resulted in sufficient coverage of the oil-water interface, leading to larger droplets (Tadros et al., 2004); higher concentrations improved surface coverage and stability by facilitating particle attachment to the interface (Yan et al., 2024).

At fixed MFGM-CP concentrations, droplet size increased significantly (*P* < 0.05) with higher oil phase ratio at low concentrations (c = 0.5%, 1%), while no variation occurred at 2% concentration. This aligns with Mwangi et al.'s finding on chitosan-stabilized Pickering emulsions (Mwangi et al., 2016), suggesting limited agglomeration at higher oil phase rations due to reduced available particles (Leal-Calderon & Schmitt, 2008). Given that all MFGM-CP stabilized emulsions were oil-in-water (O/W) emulsions, their droplet diffusion rates in deionized water slowed as oil phase ratio increased.

### 3.3 Emulsification properties of MCP emulsions

The emulsification activity index (EAI) and stability index (ESI) reflect the emulsions' properties. Fig. 1D-F displays EAI and ESI for varying MFGM-CP complex concentrations (0.5–4%, *w*/*v*) and oil-phase ratios (0.1–0. 3). When ϕ = 0.3, increasing MFGM-CP concentration decreased EAI from 15.43 ± 0.83 m^2^/g to 4.49 ± 0.14 m^2^/g, while ESI increased from 45.78 ± 2.06 min to 2673.57 ± 175.07 min. This is consistent with finding using bovine gelatin (Zhang, et al. 2020) and regenerated filipin protein (Huang et al., 2023). Lower concentration emulsions exhibit diffusion - controlled protein adsorption at oil-water interface, while higher concentrations prevent diffusion-related mobility, allowing for more proteins to remain in the aqueous phase (Gimenez et al., 2009). Increased protein-protein interactions at higher concentrations may also lower oil-water interface protein levels, reducing EAI and enhancing ESI (Nagarajan et al., 2012).

At fixed particle concentration, EAI decreased with rising oil volume, while ESI initially decreased at c = 0.5–1%, but increased at c = 2–4%. This behavior arises because low concentration complex cannot adequately cover oil droplets during homogenization leading to instability (Aziz et al., 2020). Pectin addition significantly enhanced ESI compared to the 2% MFGM stabilized emulsions (*P* < 0.05), similar to the effect of dandelion polysaccharides in whey isolate protein emulsion (Han et al., 2024). Liang et al. noted that polysaccharides improved protein adsorption and stabilization at the oil-water interface under acidic conditions (Liang et al., 2023).

### 3.4 Determination of viscosity properties of MCP emulsions

**Fig. 3:**
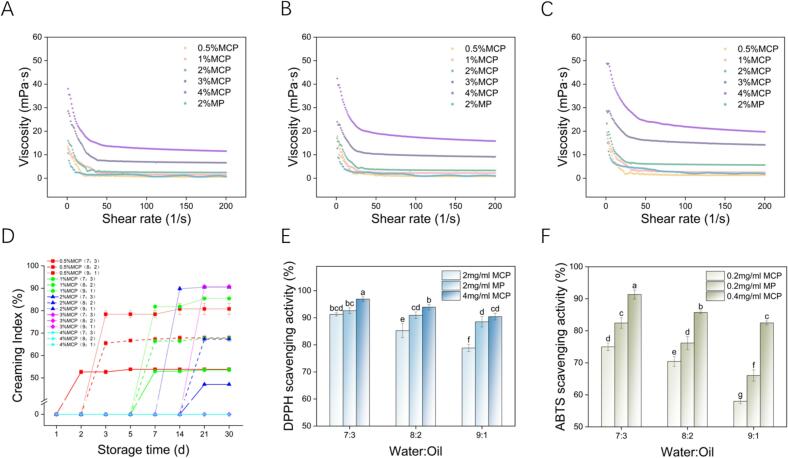
Viscosity scanning curve of emulsions at varying concentrations of MFGM-CP complex (c, 0.5–4%, w/v) and oil phase ratios (ϕ, 0.1–0.3). (A-C) apparent viscosity; (D) creaming index; (E) DPPH-radical scavenging ability; (F) ABTS-radical scavenging ability of emulsions at different oil phase ratios. Different letters indicate significant differences (*P* < 0.05).

Viscosity indicates emulsion fluidity, with higher viscosity generally correlating with improved stability (Zhang et al., 2024). Fig. 3A-C illustrates viscosity changes with shear rate in emulsions of varying MFGM-CP complex concentrations and oil phase ratios. Emulsion viscosity increased with MFGM-CP complex concentration and oil phase ratio. The addition of pectin significantly changed the viscosity of emulsions, while emulsion viscosity decreased with shear rate. This trend occurs as high shear rates disrupted emulsion structure, reducing viscosity. Emulsions with high oil content or particle density exhibit higher resistance to shear stress due to greater packing density and reduced interparticle voids (Wei & Huang, 2019).

### 3.5 Storage stability of MCP emulsions

**Fig. 4:**
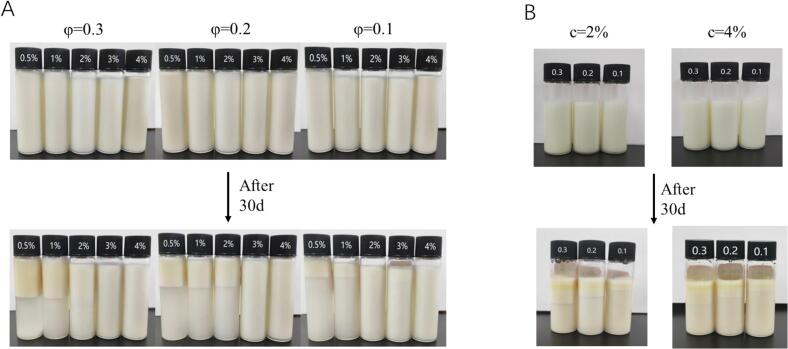
Emulsion visual observation plots. (A) Emulsions after 28 days of storage at varying concentrations of MFGM-CP complexes (c, 0.5–4%, w/v) and oil phase ratios (ϕ, 0.1–0.3); (B) Emulsions stabilized by 2% and 4% MFGM after 28 days of storage.

The creaming index (CI) measures the rate of delamination and the ratio of separated aqueous phase to total emulsion; lower CI indicate more homogeneous and stable systems (Faria Freitas et al., 2020). Following 30 days of storage, emulsion exhibited varying degrees of phase separation (Fig. 4A). With a fixed oil phase volume, increased MFGM-CP concentration gradually inhibited phase separation, with complete suppression observed at 4% concentration (Fig. 3D). High MFGM-CP concentrations enhance continuous phase viscosity and strengthen the Pickering emulsion network (Lin et al., 2022), similar to findings with Ovo transferrin-lysozyme particles (Wei et al., 2019), whey protein-chitosan particles (Lv et al., 2020), and soy protein particles (Liu & Tang, 2013). At a complex concentration of 3%, emulsion with ϕ = 0.1 showed delamination, while those with ϕ = 0.2, 0.3 remained stable, attributed to increased viscoelasticity inhibited the droplet flow (Liu et al., 2021). Notably, pectin enhanced the MFGM's emulsifying capacity, as MFGM stabilized emulsions (c = 2%, 4%) experienced deterioration with oil removal after 30 days (Fig. 4B). Pectin addition significantly bolstered nanofibrillated egg white protein emulsion stability (Alavi & Chen, 2022). Phospholipids, as the backbone of MFGM, are multifunctional amphipathic molecules with excellent emulsifying properties; their structure reduces interfacial tension between oil and water, enhancing emulsion stability (Pan et al., 2024).

### 3.6 Oxidative stability of MCP emulsions

**Fig. 5:**
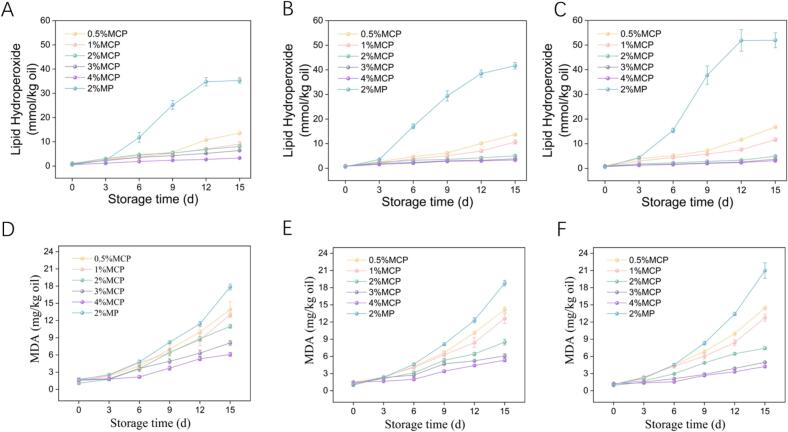
Peroxide (A-C) and 2-thiobarbituric acid (D—F) values of emulsions at varying MFGM-CP complex concentrations (c, 0.5–4%) and oil phase ratios (ϕ, 0.1–0.3).

Due to the susceptibility of vegetable oils to oxidation in aerobic conditions, the oxidative stability of rice oil in MFGM-CP-stabilized emulsions was assessed. Lipid hydroperoxide (LH) and malondialdehyde (MDA) levels were measured in emulsions with varying complex concentrations (c, 0.5–4%, *w*/*v*) and oil-phase ratios (ϕ, 0.1–0.3) over 0–14 days at 50 °C, as illustrated in Fig. 5. LH and MDA levels were inversely related to the oil-water ratio throughout the measurement period. The reduction in the LH and MDA values as the MFGM-CP concentration increased from 0.5% by 4% is attributed to a denser interfacial adsorbent layer at the oil-water interface, limiting oxygen penetration, thereby enhancing oxidative stability (Berton-Carabin et al., 2014).

On day 14, the emulsion stabilized by 2% MFGM exhibited an LH value of 35.31 ± 1.17 mmol/kg oil and an MDA value of 17.85 ± 0.52 mg/kg oil. The incorporation of 2% CP reduced LH (3.32 ± 0.07 mmol/kg oil) and MDA (6.09 ± 0.34 mg/kg oil), suggesting that CP addition retarded lipid oxidation and contributed to a network structure that effectively binds oil droplets (Abdollahi et al., 2020).

### 3.7 Centrifugal stability of MCP emulsions

**Fig. 6:**
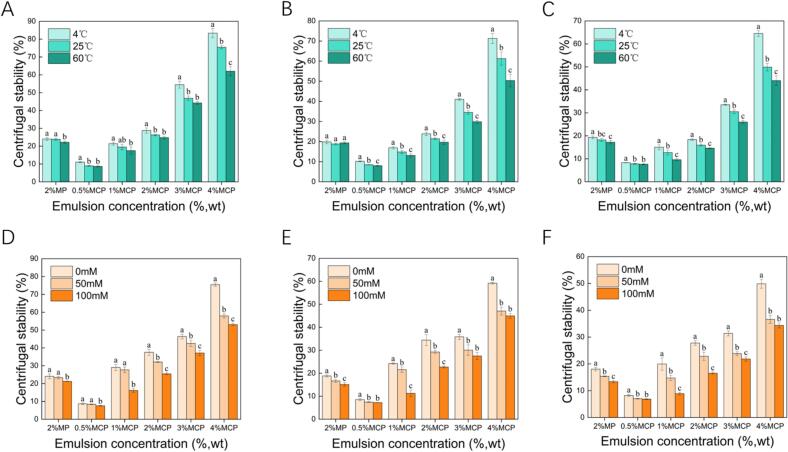
Centrifugal stability of emulsions stabilized by varying concentrations of MFGM-CP complexes (c, 0.5–4%) and oil-phase ratios (ϕ, 0.1–0.3) across different temperatures (A-C) and salt ion concentrations (D—F). Different letters denote significant differences (*P* < 0.05).

Higher centrifugal stability correlates with greater emulsion stability in various environments. Thus, we examined the centrifugal stability of emulsions prepared with diverse particle concentrations (c, 0.5–4%, w/v) and oil-phase ratios (ϕ, 0.1–0.3) at varied temperatures (4 °C, 25 °C, and 60 °C) and salt ion strengths (0 mM, 50 mM, and 100 mM) (Fig. 6A-C). Centrifugal stability increased with MFGM-CP concentration at a specific oil - phase ratio. Emulsion at 4 °C demonstrated significantly higher than at 25 °C and 60 °C (*P* < 0.05) due to decreased thermal motion, reducing droplet aggregation and enhancing stability at lower temperature (Li et al., 2022).

Salt ions reduce the surface potential of colloidal particles, leading to flocculation (Yan et al., 2024) (Fig. 6D-F). The centrifugal stability of MCP emulsions generally decreased with higher salt ion concentrations. Similarly, Song et al. reported that increasing salt ion concentrations in Pickering emulsion stabilized by buckwheat protein colloidal particles resulted in increased droplet size, from 4.2 ± 0.1 μm to 29.0 ± 1.0 μm with a concentration rise from 0 mmol/L to 300 mmol/L (Song et al., 2023). High salt concentration affects the electrostatic repulsive force and spatial interactions between MFGM-CP particles, weakening the interfacial film strength and facilitating droplet aggregation, thereby reducing centrifugal stability (Yan et al., 2024). Holding particle concentration constant, centrifugal stability declined as oil content increased, following the same trend as the EAI of the emulsion. Likewise, pectin addition improved centrifugal stability due to increasing emulsion viscosity, which mitigated phase separation during centrifugation (Dong et al., 2023).

### 3.8 ABTS and DPPH free radical scavenging capacity of the MCP emulsions

Because oxidation diminishes probiotic activity during processing and storage, the antioxidant activity of MFGM-CP emulsions was evaluated via DPPH⋅ and ABTS+ scavenging activities (Fig. 3E-F). The DPPH⋅ scavenging capacity of emulsions escalated from 78.83 ± 1.37% to 91.25 ± 0.66% at a concentration of 2 mg/mL as ϕ value rose from 0.1 to 0.3. At 4 mg/mL, the DPPH⋅ scavenging capacity of MCP emulsion at ϕ = 0.3 reached 96.88 ± 0.85%, significantly surpassing that at 2 mg/mL (*P* < 0.05). The ABTS+ scavenging capacity mirrored the DPPH⋅ results, indicating both are concentration and oil - phase ratio dependent. This dependency arises because increased emulsion oil phase or complex concentration encapsulates more free oil droplets creating a robust mesh structure that obstructs free radicals movement and oxygen entry (Zhao et al. 2023). Notably, the free radical scavenging ability of the emulsion with 2% MFGM exceeded that of the 1% MFGM and 1% CP combination (*P* < 0.05), yet was lower than that of the 2% MFGM and 2% CP emulsion (*P* < 0.05), underscoring CP's role in enhancing antioxidant capacity, predominantly attributed to MFGM.

### 3.9 Storage stability and stability in simulated gastrointestinal digestion of LGG in MCP emulsion

**Fig. 7:**
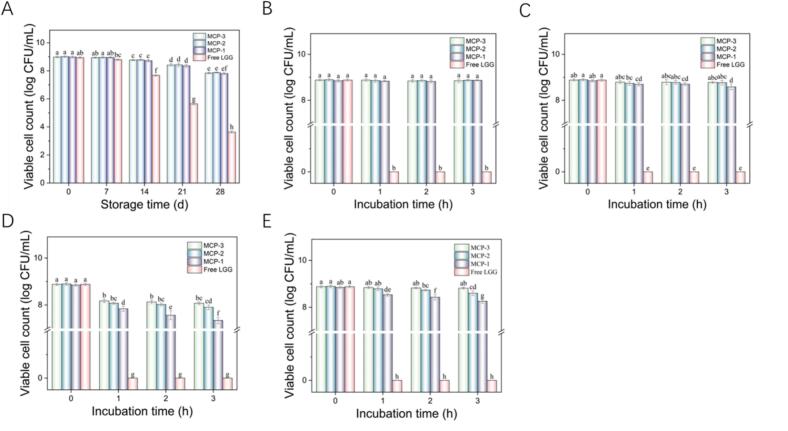
Viable cell counts of LGG in water and 4% MCP emulsions with varying oil phase ratios (ϕ, 0.1–0.3): (A) stored for 28 days; (B—D) in simulated gastric fluid at pH 2.0, 1.8, and 1.5; (E) in simulated intestinal fluid. Different letters denote significant differences (*P* < 0.05).

**Fig. 8:**
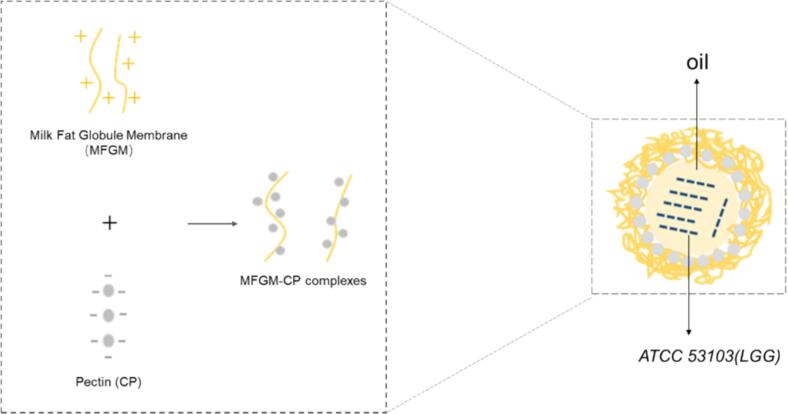
Schematic representation of the process of binding of MFGM and CP by electrostatic interaction and emulsion-embedded probiotic bacteria formed by MFGM-CP complexes.

Maintaining probiotic activity throughout and storage is vital. Thus, this study selected emulsion-embedded probiotics with varying ϕ values and a minimal CI (c = 4%) for additional experiments. As shown in Fig. 7A, after 28 days of storage at 4 °C, viable LGG counts in both water and emulsions decreased with extended storage (*P* < 0.05). However, compared to free LGG, LGG encapsulated by MCP emulsion significantly enhanced survival rates during long-term storage (*P* < 0.05) with no significant difference among emulsions with different ϕ values. Emulsions create a protective barrier around probiotics, shielding them from environment influences and sustaining a stable survival environment (Liang et al., 2023). Antioxidant components in the MFGM-CP emulsions scavenge harmful free radicals and shield probiotics from oxidative stress (Guo et al., 2022). Furthermore, MFGM and CP also serve as prebiotics, providing essential nutrients for probiotic survival and extending viability within the emulsion (Manthei et al., 2024; Yang et al., 2024). Fig. 8 illustrates the structure of the probiotic delivery system constructed from the MFGM-CP complex, which encapsulates the probiotic active ingredient within an oil-phase core while forming an aqueous-phase protective layer composed of MFGM-CP complex on the outer layer.

Preserving probiotic survival in the gastrointestinal tract is essential for their functionality. The natural pH of gastric juice ranges from 1.5 to 3.5. This study tested free and encapsulated LGG in simulated gastric juice at pH 2.0, pH 1.8, and pH 1.5 over 0–3 h. Results (Fig. 7B-D) demonstrated that all free LGG perished within one hour across pH conditions. Conversely, MCP emulsion embedding significantly improved probiotic survival. LGG survival rate decreased with lower pH and longer exposure times, yet with the same pH and duration, survival rate was notably high in emulsions with higher oil phase ratios. In pH 1.5 gastric fluid after 3 h, the viable cell counts at ϕ = 0.3 (8.88 ± 0.05 log CFU/mL) surpassed that at ϕ = 0.1 (7.34 ± 0.14 log CFU/mL) (*P* < 0.05).

In simulated intestinal fluids, MCP emulsions also showed enhanced protective effects at elevated ϕ values (Fig. 7E). This observation is consistent with findings by Qin et al. when encapsulating *L. plantarum* with WPI-EGCG (Qin, et al. 2021). Higher ϕ values led to emulsions less prone to degradation by digestive enzymes, achieved slower digestion rates, and consequently improved encapsulated LGG survival (Wan et al., 2020). Additionally, nanofibers created via electrostatic spinning with MFGM-pullulan were found to bolster LGG tolerance to the gastrointestinal environment (Wang et al., 2024).

Phospholipids are essential components of probiotic cell membranes and promote probiotic growth and reproduction. Studies indicate that phospholipids in MFGM enhance the vitality of probiotics in the gut and facilitate their colonization (Yao et al., 2023). They can either interact with probiotics via hydrophobic mechanisms to prolong their stability during storage (Plaza et al., 2023) or enhance bacterial surface hydrophobicity, altering fatty acid composition in cell membrane, and improving probiotic viability in high - bile salt environments (Hu et al., 2015). Furthermore, phospholipids exhibit free radical scavenging capabilities that mitigate oxidative stress damage to probiotics, thereby improving their antioxidant capacity (Ali et al., 2020).

This study examined the stability characteristics of the MFGM-CP emulsion system across various concentration gradients (0.5–4.0%, *w*/*v*) and oil-phase ratios (ϕ, 0.1–0.3) to identify optimal carrier concentrations for probiotic encapsulation. We evaluated the emulsion system's ability to maintain the long-term viability of probiotics and survival in a simulated gastrointestinal environment. However, this research did not assess the long-term storage effects or potential changes in the emulsion's sensory properties, necessitating future studies to systematically explore the sensory transformations during extended storage.

## 4 Conclusion

This study demonstrated that the emulsion formed by Milk fat globule membrane (MFGM) and Pectin (CP) via electrostatic interactions constitute an effective delivery system for enhancing probiotic viability under simulated gastrointestinal conditions and prolonged storage. MFGM-CP Pickering (MCP) emulsions exhibited optimal storage and oxidative stability at a MFGM-CP concentration of 4% and an oil phase ratio of 0.3. The interaction between MFGM and CP enhances both the centrifugal stability and antioxidant activity of MCP compared to MFGM-stabilized emulsions. In conclusion, the electrostatic interaction between MFGM and CP enhances the properties of Pickering emulsions, facilitating the formation of lipid-containing protein-polysaccharide complexes, and offering a novel strategy for the protection of probiotic bacteria. Our findings suggest that these results may be applied to encapsulate and improve the stability of infant probiotics.

## CRediT authorship contribution statement

**Yu Ji:** Writing – original draft, Methodology, Data curation. **Yifan Wu:** Formal analysis, Data curation. **Yan Wang:** Software, Formal analysis. **Shuangshuang He:** Visualization, Software. **Yishan Jiang:** Validation, Conceptualization. **Xin Li:** Visualization, Methodology. **Liupeng Wang:** Visualization, Software. **Qian Xu:** Investigation. **Lili Zhang:** Writing – review & editing, Supervision, Resources, Project administration, Funding acquisition, Conceptualization. **Hao Wang:** Resources, Project administration, Conceptualization.

## Declaration of competing interest

The authors declare that they have no known competing financial interests or personal relationships that could have appeared to influence the work reported in this paper.

## Data Availability

Data will be made available on request.
